# A Method of Handling Alveolar Pyorrhea.—Abstract

**Published:** 1898-10

**Authors:** Geo. T. Carpenter

**Affiliations:** Chicago


### A Method of Handling Alveolar Pyorrhea.—Abstract.

Read before the Stomatological Section of the American Medical Association at Denver, June, 1898.

BY GEO. T. CARPENTER, M.D., D.D.S. OF CHICAGO.

In presenting this paper before this section, I do so with a feeling that the practical side should not be lost sight of while we are eagerly searching out the scientific. It is not my purpose in this paper to discuss the various points above mentioned, but to give a brief outline or method of handling these cases which has given me the most satisfactory results. It has been my experience that different cases of pyorrhea differ in their treatment. One case may yield readily to a certain treatment while another may be very persistent in its pathological behavior. But thoroughness in diagnosis and treatment is the key to success. In diagnosing any case, get a history of it and decide on the predisposing cause, or causes outside the pocket which is generally from local irritation caused by wooden tooth-picks, ligature, wedging of teeth, ill-fitting plates or crowns accompanied with neglect of the mouth. The disease once established is self-sustaining and it is within the pocket that we must look for, find and remove the exciting cause, which may be serumal deposits, pus secreting membrane, foreign substances or diseased or necrosed margins of process. To make a thorough and careful examination of the pockets I secure dryness with bibulous paper and protect the parts with a napkin and coat the surrounding gum with cosmoline to protect the parts outside of the pocket from the action of medicines. Now dry the pocket and pack with cotton slightly moistened with four per cent. to eight per cent. aqueous solution of cocaine. After allowing this to remain several minutes, carefully remove the cotton. The pocket will be found to remain open so that an examination can be made with a mouth mirror. Deposits if present can be located before instruments are used for their removal. I operate on but one tooth at a time, or on the interproximal space between two teeth, doing all that I intend to do in that immediate locality before directing my attention to other teeth. All loose teeth having lost two-thirds or more of their natural support are extracted. The best instruments to use in the pockets are spoon shaped excavators with the shanks bent so as to reach every part of the root or pocket. The soft parts and margin of process can also be curetted if necessary. The pocket should be cleansed with equal parts of per oxide of hydrogen and pasteurine, or three per cent. pyrozone and borolyptol, using a flat-pointed syringe and pass to the bottom of the pocket. This syringe is designed especially to reach pyorrhea pockets and can be used as successfully on posterior teeth as in the front part of the mouth and fluids do not escape beside the point until the pocket is distended and all parts are reached. This will not only antiseptically cleanse, but mechanically carry out all free and loosened particles. The gums are now wiped dry and painted with tincture of iodine, which should be repeated about twice a week. The tooth, if loose, is supported by bands or ligatures, and if moved by occlusion I grind the tooth, or its antagonist, sufficient to prevent all movement, so as to procure rest for the affected parts. All subsequent treatment of the pocket should be made by the use of a thinly shaved hard wood tooth-pick, which can be moistened in the antiseptic and bent so as to reach any location. This should be passed into the pocket as deep as possible without injury to the tissue, or causing pain or hemorrhage; and repeat the application once or twice a week, as long as the tooth-pick can gain entrance to the pocket. It is seldom, if ever, necessary to make incisions through the gums into the pocket and I but seldom resort to the use of dilute or stronger acids as an aid in softening or removing deposits or to establish resolution. Antiseptic and prophylactic conditions should be established and followed during treatment, and indefinitely continued throughout life. I recommend a thorough rinsing of the mouth and the use of an antiseptic after each meal. I also recommend a medium stiff tooth brush to be carefully used once in 24 hours, and that at night before retiring. This time, once established, will not be substituted for other hours in the day. The brush will have time to dry, and the elasticity of bristles restored, which will do the best work at the most needed time. A good dentrifice with the best precipitated chalk should be used on the brush, after which one drachm of pasteurine or borolytol should be taken into the mouth and retained, for from three to five minutes, at the same time moving the cheeks and lips so as to force it between the teeth and reach all parts of the mouth, then expectorate and retire without rinsing the mouth with water. The time spent in the successful treatment of this disease depends on the constitutional condition of the patient and the extent of the disease, or the thoroughness in which the first operation is performed. The recurrence of the disease comes from a failure to remove the predisposing causes. The reproduction of gum tissue in all interproximal spaces is essential to prevent the lodgment of food or injudicious use of the tooth-pick. I believe that a thorough removal of the cause, with antiseptic treatment and stimulation of the gum tissue will cure the most persistent case of alveolar pyorrhea.

